# The potential role of scavenger receptor B type I (SR‐BI) in SARS‐CoV‐2 infection

**DOI:** 10.1002/iid3.786

**Published:** 2023-04-14

**Authors:** Luay Alkazmi, Hayder M. Al‐kuraishy, Ali I. Al‐Gareeb, Athanasios Alexiou, Marios Papadakis, Hebatallah M. Saad, Gaber El‐Saber Batiha

**Affiliations:** ^1^ Biology Department, Faculty of Applied Sciences Umm Al‐Qura University Makkah Saudi Arabia; ^2^ Department of Clinical Pharmacology and Medicine College of Medicine ALmustansiriyia University Baghdad Iraq; ^3^ Department of Science and Engineering Novel Global Community Educational Foundation Hebersham New South Wales Australia; ^4^ AFNP Med Wien Austria; ^5^ Department of Surgery II University Hospital Witten‐Herdecke University of Witten‐Herdecke Wuppertal Germany; ^6^ Department of Pathology Faculty of Veterinary Medicine Matrouh University Matrouh Egypt; ^7^ Department of Pharmacology and Therapeutics Faculty of Veterinary Medicine Damanhour University Damanhour Egypt

**Keywords:** COVID‐19, pro‐inflammatory cytokines, scavenger receptor type B I

## Abstract

Scavenger receptor type B I (SR‐BI), the major receptor for high‐density lipoprotein (HDL) mediates the delivery of cholesterol ester and cholesterol from HDL to the cell membrane. SR‐BI is implicated as a receptor for entry of severe acute respiratory syndrome coronavirus type 2 (SARS‐CoV‐2). SR‐BI is colocalized with the angiotensin‐converting enzyme 2 (ACE2) increasing the binding and affinity of SARS‐CoV‐2 to ACE2 with subsequent viral internalization. SR‐BI regulates lymphocyte proliferation and the release of pro‐inflammatory cytokines from activated macrophages and lymphocytes. SR‐BI is reduced during COVID‐19 due to consumption by SARS‐CoV‐2 infection. COVID‐19‐associated inflammatory changes and high angiotensin II (AngII) might be possible causes of repression of SR‐BI in SARS‐CoV‐2 infection. In conclusion, the downregulation of SR‐BI in COVID‐19 could be due to direct invasion by SARS‐CoV‐2 or through upregulation of pro‐inflammatory cytokines, inflammatory signaling pathways, and high circulating AngII. Reduction of SR‐BI in COVID‐19 look like ACE2 may provoke COVID‐19 severity through exaggeration of the immune response. Further studies are invoked to clarify the potential role of SR‐BI in the pathogenesis of COVID‐19 that could be protective rather than detrimental.

## Graphical abstract

We illustrate that Scavenger receptor type B I (SR‐BI), the major receptor for high‐density lipoprotein (HDL) mediates delivery of cholesterol ester and cholesterol from HDL to the cell membrane. SR‐BI is implicated as a receptor for entry of severe acute respiratory syndrome coronavirus type 2 (SARS‐CoV‐2). Downregulation of SR‐BI in COVID‐19 could be due to direct invasion by SARS‐CoV‐2 or through upregulation of pro‐inflammatory cytokines, inflammatory signaling pathways and high circulating AngII.

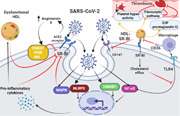

## 1 BACKGROUND

Scavenger receptor (SR) was first identified in 1970 by Brown and Goldstein as a receptor of oxidized low‐density lipoprotein (oxLDL) that promotes the development of foam cells from activated macrophages and is linked with the pathogenesis of atherosclerosis.^1^ SRs involve different integral membrane proteins and extracellular domains that are ligands for lipoproteins, phospholipids, cholesterol esters, apoptotic cells, proteoglycans, carbohydrates, and ferritin.^1^ Later on, SRs were classified from A‐J according to their biological functions and structures.^2^

**Figure 1:**
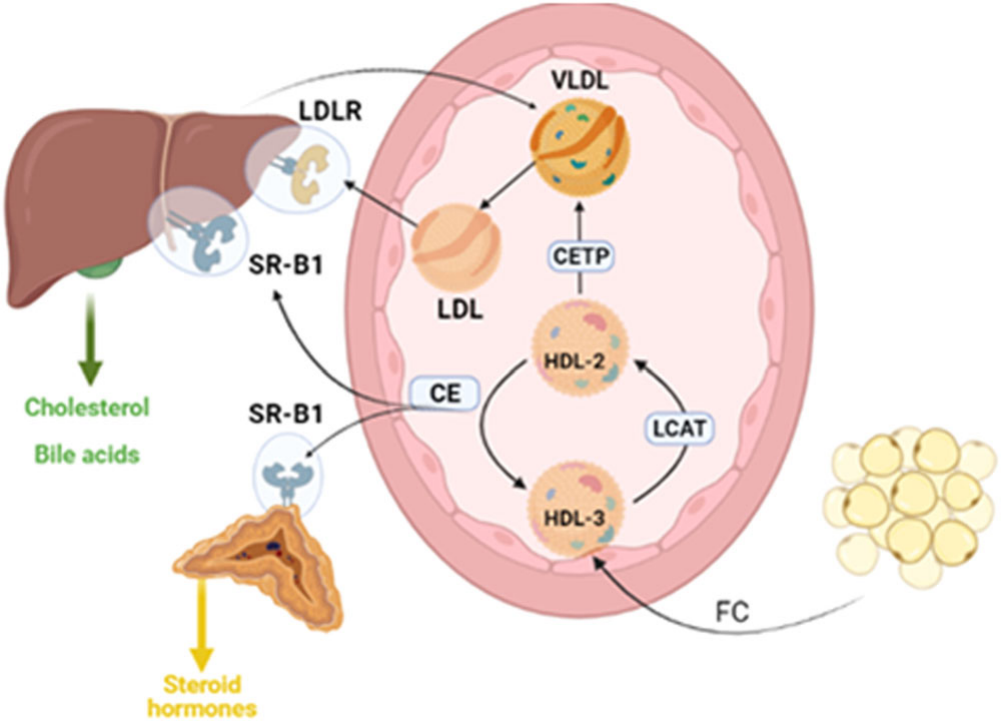
The biological role of scavenger receptor type B I (SR‐BI). Free cholesterol (FC) from peripheral tissues bind to high‐density lipoprotein (HDL), through lecithin cholesterol acyl transferees (LCAT) and cholesterol ester transferase protein (CETP) form very‐low‐density lipoprotein (VLDL). HDL through SR‐BI improves steroidogenesis in the adrenal cortex and liberates cholesterol ester (CE) to the liver.

SR type B I (SR‐BI) have a trans‐membrane region, cytosolic part, and central domain which mediate ligand recognition. The central region is involved in trafficking and signal transduction.^2^ SR‐BI gene is located on chromosome 4, which is regulated by the immune response, infections, and metabolic disorders.^1^ SR‐BI also binds bacteria, viruses, and damage‐associated molecular patterns (DAMPs).^3^ SR‐BI is highly expressed in the liver and adrenal cortex that mediate cholesterol uptake for bile synthesis and steroidogenesis respectively^4^ (Figure 1).

SR‐BI is the major receptor for high‐density lipoprotein (HDL) and mediates the delivery of cholesterol ester and cholesterol from HDL to the cell membrane. Other lipids like triglycerides, phospholipids, and lipophilic molecules can also be transferred by HDL.^1^, ^2^ Selective lipid uptake depends mainly on the SR‐BI‐HDL complex since lipid‐poor HDL has low affinity to the SR‐BI and is released back into the circulation^5^ suggesting the HDL‐dependent effect for uptake of lipids through SR‐BI. Shen et al.^5^ observed that SR‐BI is regarded as a regulator of cholesterol content in the plasma membrane and promotes the uptake of fat‐soluble vitamins. In addition, SR‐BI is considered a potential site for viral entry into host cells.^5^ These pleiotropic and multiple functions of SR‐BI eventually affect vascular inflammation, platelet function, and programmed cell death.^5^, ^6^

Moreover, SR‐BI has an important role in the process of inflammation by modulating the HDL effect on fat‐induced inflammation in obesity through the regulation expression of peroxisome proliferators activated receptors (PPARs).^7^ Aldossari et al.^8^ found that SR‐BI plays an imperative role in macrophage activation by recognition of charged particles. Interestingly, SR‐BI is involved in various types of infections, it is regarded as receptor entry for mycobacterium and hepatitis C virus (HCV).^9^ However, deficiency of SR‐BI did not affect the disease severity and high cholesterol enhances bacterial and viral burdens in the lung independent of SR‐BI expression.^9^, ^10^, ^11^

**Figure 2:**
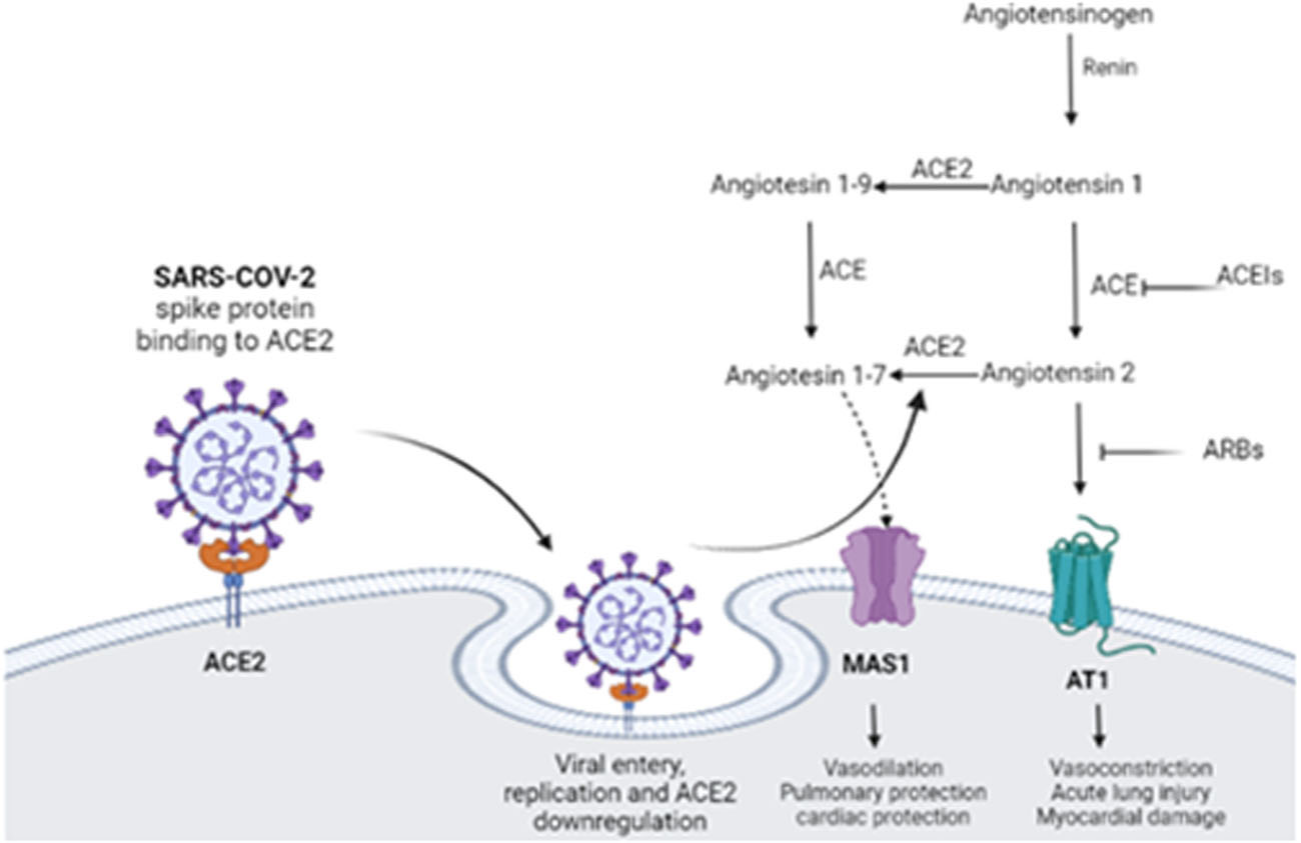
Role of the renin‐angiotensin system (RAS) in COVID‐19. severe acute respiratory syndrome coronavirus type 2 (SARS‐CoV‐2) binds angiotensin‐converting enzyme 2 (ACE2) as an entry‐point, downregulation of ACE2 induces the release of pro‐inflammatory cytokines and upregulation of angiotensin II. Angiotensin‐converting enzyme inhibitors (ACEIs) and angiotensin II receptor blockers (ARBs) increase the expression of ACE2.

A recent pandemic viral infection caused by severe acute respiratory syndrome coronavirus type 2 (SARS‐CoV‐2) leads to the development of coronavirus disease (COVID‐19).^12^, ^13^ SARS‐CoV‐2 exploits angiotensin‐converting enzyme 2 (ACE2) as an entry point. Downregulation of ACE2 induces the release of pro‐inflammatory cytokines and upregulation of angiotensin II (AngII).^14^ These changes may lead to hypercytokinemia and the development of cytokine storm with the progression of acute lung injury (ALI) and acute respiratory distress syndrome (ARDS) (Figure 2).^15^

Of interest, SARS‐CoV‐2 infection may induce dyslipidemia and the development of dysfunctional HDL due to the involvement of SR‐BI in the pathogenesis of COVID‐19.^16^ Therefore, the objective of the present review was to find the possible role of SR‐BI in SARS‐CoV‐2 infection.

## 2 ROLE OF SR‐BI IN VIRAL INFECTIONS

It has been reported that increased expression of SR‐BI is linked with higher susceptibility to HCV. Entry of HCV into the cells required SR‐BI as an entry point into host cells through a PH‐dependent mechanism.^17^ Many reports suggest that HDL can promote entry of HCV via interaction with SR‐BI, though HDL did not modulate the interaction between HCV and SR‐BI.^17^ Alteration of SR‐BI intra‐cytoplasmic domain reduces HCV infectivity without effect on the binding capacity at the extracellular domain.^17^ SR‐BI together with CD81 tetraspanin and claudin‐1 increases the penetration of HCV through endocytosis of viral particles.^17^ Therefore, antibodies directed against SR‐BI inhibit the entry of HCV in the permissive cells.^18^ Nevertheless, HCV can enter the host cells in presence of neutralizing antibodies through ApoB‐containing lipoproteins.^19^

Relevant, the influenza virus can bind pulmonary SR‐BI causing pulmonary inflammation through downregulation of lung epithelial SR‐BI, which is important for the recruitment of immune cells during inflammation.^20^ This finding suggests the protective effect of SR‐BI influenza virus‐induced pulmonary inflammation. Therefore, activation of SR‐BI could be a therapeutic modality against the severity of influenza virus infection. Though, quercetin which promotes the expression of SR‐BI^21^ is regarded as an inhibitor for entry of H5N1 and can be used as a prophylactic and therapeutic natural agent against influenza virus infection.^22^, ^23^

Moreover, the reduction of HDL and other lipoproteins are correlated with the severity of dengue hemorrhagic fever due to the activation of SR‐BI by flavivirus.^24^ SR‐BI facilitates the entry of flavivirus which interacts with ApoA1.^24^ This verdict proposed that ApoA1 drives flavivirus to interact with SR‐BI and subsequent entry. Besides, SR‐BI is also acting as a receptor for entry of the Zika virus.^25^ This observation could explain the alteration in lipid and lipoprotein metabolism in dengue patients.

These findings suggest the potential role of SR‐BI in the viral entry of different viral infections, and targeting this receptor may attenuate viral entry and infectivity.

## 3 SR‐BI AND IMMUNOLOGICAL RESPONSE

In general, SRs are cell surface proteins expressed by macrophages, monocytes, hemocytes, and other immune cells. SRs exhibit ligand binding properties, recognizing various ligand types like microbial constituents and recognition and DMRPs.^26^ SRs play an integral role in the regulation of innate immunity by acting as pattern recognition receptors (PRRs) for DMRPs mediating phagocytosis by macrophages.^27^ Some SRs are regarded as a coreceptor for the activation of toll‐like receptors (TLRs) and the release of pro‐inflammatory cytokines in response to DMRPs.^27^

SR‐BI, which has a structural similarity to CD36, is mainly expressed in the dendritic cells and macrophages.^28^ SR‐BI had been reported to increase bacterial virulence through the uptake and binding of bacterial pathogens.^29^, ^30^ Feng et al.^31^ illustrated that deficiency of SR‐BI causes impairment of lymphocyte homeostasis and induction of autoimmune disorders in experimental models. SR‐BI regulates lymphocyte proliferation and the release of pro‐inflammatory cytokines from activated macrophages and lymphocytes.^31^ Of note, SR‐BI stimulates endothelial nitric oxide synthase (NOS) in presence of HDL and induces endothelial apoptosis in the absence of HDL.^32^ As well, SR‐BI inhibits B lymphocyte proliferation and negatively regulates IgM production and cytokine production.^33^ Guo et al.^34^ demonstrated that SR‐BI may prevent septic and endotoxin‐induced death by inhibiting pro‐inflammatory cytokine production from macrophages.

Concerning the imperative role of SR‐BI in adaptive immune response, different studies revealed that deficiency of SR‐BI enhances lymphocyte proliferation, the release of pro‐inflammatory cytokines and imbalance of interferon‐gamma (INF‐γ) production in relation to the anti‐inflammatory cytokine interleukin 4 (IL‐4) with 3‐4 fold increase of activated B and T lymphocytes.^31^ These changes due to deficiency of SR‐BI trigger autoimmune disturbance with the elevation of circulating autoantibodies and deposition of immune complexes in renal glomeruli.^31^

**Figure 3:**
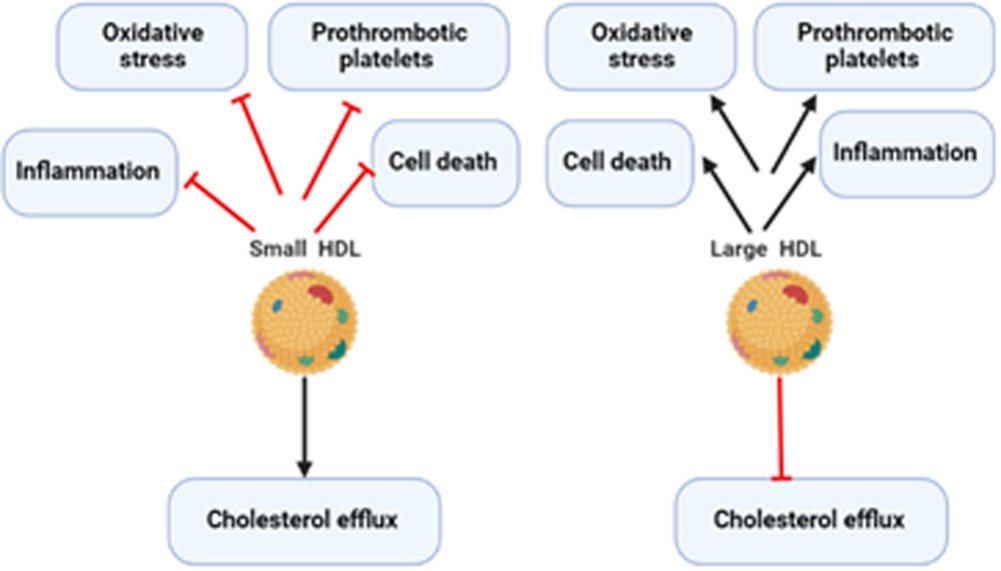
High‐density lipoprotein (HDL)‐dependent effect of scavenger receptor type B I (SR‐BI).

Zhu and colleagues found that deficiency of SR‐BI attenuates the suppressant effect of HDL on the lymphocytes and the expression of TLR9 which enhances B lymphocyte proliferation.^33^ Entertainingly, SR‐BI inhibits macrophage activation by suppressing TLR4 and NF‐κB, thus a deficiency of SR‐BI provokes macrophage activation and release of pro‐inflammatory cytokines.^35^, ^36^ Indeed, SR‐BI regulates lymphocyte cellular cholesterol content which controls lymphocyte activation.^31^ Similarly, Zheng et al.^37^ illustrated that SR‐BI maintains T cell development and thymic regeneration. It has been shown that thymic involution impairs T cell development and adaptive immune response that increases the risk of various types of infections.^38^ Likewise, small HDL via stimulation of SR‐BI produces protective effects against inflammation, oxidative stress, and cell death.^39^ Though, large HDL via inhibition of SR‐BI induces detrimental effects on inflammation and oxidative stress.^31^ This finding suggests the HDL‐dependent effect of the protective SR‐BI (Figure 3).

Taken together, these findings anticipated that SR‐BI has an immunoregulatory function by modulating B and T lymphocytes functions as well as macrophage function causing inhibition of the release of pro‐inflammatory cytokines and the development of autoimmune disorders.

## 4 SR‐BI AND ACUTE LUNG INJURY (ALI)

SR‐BI is highly expressed in lung alveolar cells involved in the regulation of lung innate immune response and glucocorticoid‐induced lymphocyte apoptosis.^40^, ^41^ A recent experimental study illustrated that lung SR‐BI attenuates lipopolysaccharide (LPS)‐induced ALI in mice through increasing clearance of LPS.^42^ Baranova et al.^43^ showed that variant SR‐BI increases the risk of LPS‐induced acute kidney injury. Moreover, short and long‐term exposure to ozone increases the risk of pulmonary inflammation and ALI via the generation of DAMPs which recognize PRRs like SRs and TLR4 in lung macrophages.^44^ SR‐BI can reduce ozone‐induced pulmonary inflammation and ALI by enhancing the clearance of DAMPs and promoting lung alveolar macrophage efferocytosis.^44^, ^45^ This finding proposes a protective role of SR‐BI against ozone‐induced ALI.

Moreover, long‐term human exposure to the ozone or cigarette smoke downregulates the expression of lung alveolar SR‐BI and reduction of vitamin E with increased susceptibility to the risk of oxidative stress injury and exacerbation of underlying chronic pulmonary disorders.^46^ SR‐BI is necessary for the transport of vitamin E from plasma into the cells and deficiency of SR‐BI increases vitamin E plasma levels twofold with a reduction of its concentrations in the tissues mainly in the lungs.^47^ Likewise, SR‐BI increases intestinal absorption of vitamin E^48^ and regulates its biliary secretion and enterohepatic absorption.^48^ Thus, vitamin E deficiency increases the expression of SR‐BI.^49^

**Figure 4:**
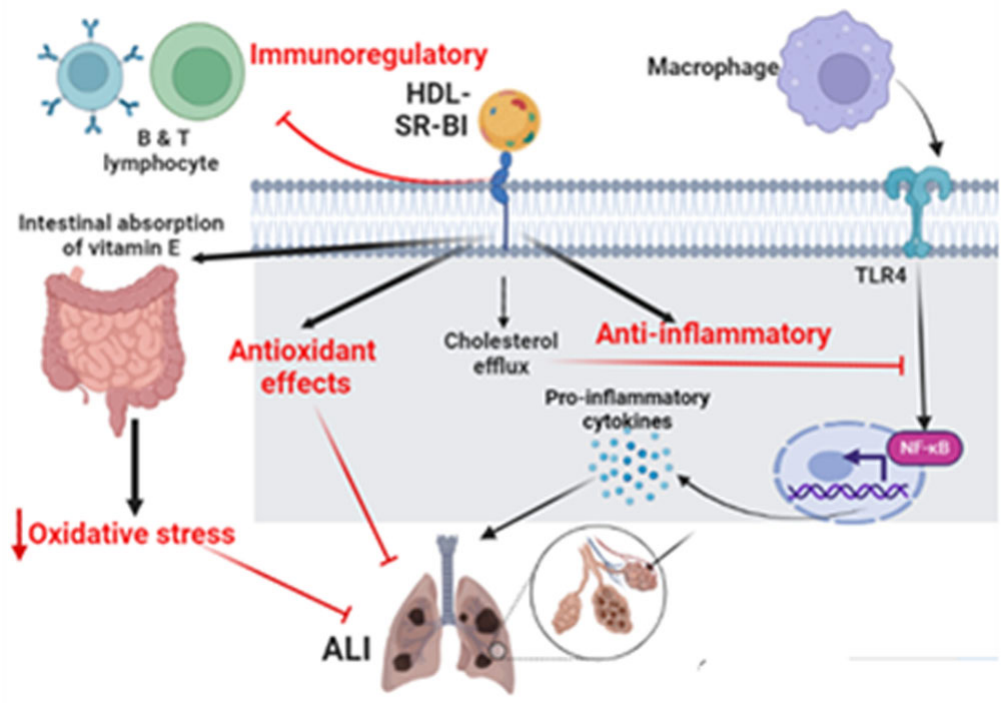
Role scavenger receptor type B I (SR‐BI) in acute lung injury (ALI) and immunoregulation. SR‐BI through antioxidant and anti‐inflammatory effects suppress the release of pro‐inflammatory cytokines and oxidative stress, thereby inhibiting the development of acute lung injury (ALI). SR‐BI modulates B and T lymphocyte functions as well as macrophage function.

Notoriously, cigarette smoke reduces the expression of SR‐BI through the induction of p38 mitogen‐activated protein kinase (p38MAPK) and the Akt pathway which inhibits the expression of SR‐BI.^50^ Cigarette smoke‐induced oxidative stress can modulate the functional capacity to take vitamin E, with subsequent reduction of lung alveolar vitamin E concentration.^51^ Of note, CD36 and SR‐BI share a similar homology and ligands mainly for oxLDL, which reduce the binding of HDL to SR‐BI, which reduces HDL‐mediated effects. Of interest, oxLDL and other mediators of oxidative stress deviate from the accumulation of modified lipoproteins in the macrophages via CD36.^52^ Lei et al.^53^ revealed that oxidative stress is regarded as an important factor in the development of ALI. These observations suggest that downregulation of lung SR‐BI due to different reasons may increase the risk of ALI and the progression of severe inflammatory changes (Figure 4).

## 5 SR‐BI AND COVID‐19

Alveolar SR‐BI plays an imperative role in the entry of SARS‐CoV‐2 since silencing of SR‐BI by a specific antagonist inhibits entry of SARS‐CoV‐2.^54^, ^55^, ^56^ HDL may facilitate entry of SARS‐CoV‐2 through activation of SR‐BI.^55^ It has been demonstrated that the receptor‐binding domain (RBD) of SARS‐CoV‐2 directly interacts with HDL, so the antibody against the HDL‐binding site reduces SARS‐CoV‐2 entry.^57^ Therefore, SARS‐CoV‐2 may bind SR‐BI directly or indirectly through interaction with HDL. To the point, SR‐BI is colocalized with ACE2 which increases SARS‐CoV‐2 binding and affinity to the ACE2 with subsequent viral internalization.^57^, ^58^ SR‐BI promotes the cholesterol content of cells and the formation of lipid rafts, which could enhance SARS‐CoV‐2 infectivity.^59^

Depletion of cell membrane cholesterol may inhibit SARS‐CoV‐2 infection^60^ therefore; cholesterol‐lowering drugs like statins may reduce SARS‐CoV‐2 infectivity.^60^ Wei and colleagues proposed that inhibition of SR‐BI may reduce HDL‐mediated SARS‐CoV‐2 entry, while overexpression may increase SARS‐CoV‐2 entry and infectivity.^61^ However, the interaction between SARS‐CoV‐2 and SR‐BI is mediated and enhanced by the presence of ACE2 since SARS‐CoV‐2 affects SR‐BI only in tissues with higher expression of ACE2 like the lung.^61^ The interaction between SARS‐CoV‐2 and SR‐BI are not well defined and further studies are needed to confirm this relationship. In the lower respiratory tract mainly at alveolar cells, both SR‐BI and ACE2 are highly expressed^62^ which increases the risk of COVID‐19 pneumonia.

Remarkably, ACE2 is highly expressed in patients with obesity and metabolic syndrome increases the risk for COVID‐19.^63^, ^64^ Likewise, SR‐BI is highly expressed in patients with hypertension and cardiovascular diseases.^65^ Therefore, higher expression of SR‐BI and ACE2 in cardio‐metabolic disorders may increase the risk of SARS‐CoV‐2 infectivity and COVID‐19 severity.^55^ Thus, targeting SR‐BI could be a promising therapeutic strategy to reduce SARS‐CoV‐2 since SR‐BI antagonist ITX5601 an approved agent for HCV, powerfully attenuates SARS‐CoV‐2 entry in vitro.^55^ However, ITX5601 increases the level of HDL^66^ which is regarded as a potential site for the binding of SARS‐CoV‐2.

Importantly, SR‐BI hampers macrophage activation through restrain of TLR4 and NF‐κB, preventing macrophage activation and release of pro‐inflammatory cytokines.^35^, ^67^ Abnormal immune response and dysregulated interferon in COVID‐19 trigger the development of macrophage activation syndrome (MAS).^68^ In turn, MAS provokes the release of pro‐inflammatory cytokines and multiorgan injury. Therefore, activation of SR‐BI may inhibit the development of MAS in COVID‐19.

SR‐BI like ACE2 is protective rather than harmful in COVID‐19; it has been shown that recombinant ACE2 is of therapeutic value in the management of COVID‐19.^69^ SR‐BI is not ultimately increasing SARS‐CoV‐2 entry unless ACE2 is present, and like ACE2, SR‐BI binds but does not internalize SARS‐CoV‐2 entry. Therefore, overexpression of SR‐BI could be protective by neutralization of SARS‐CoV‐2, since SR‐BI has a potent neutralizing effect against the influenza virus.^20^ Consumption and exhaustion of SR‐BI during SARS‐CoV‐2 infection contribute to the development of dyslipidemia which is linked with COVID‐19 severity.^16^ These elusive findings implicate SR‐BI in the pathogenesis of SARS‐CoV‐2 infection, and further studies are recommended to confirm the protective effect of SR‐BI in COVID‐19.

## 6 SR‐BI AND INFLAMMATORY SIGNALING PATHWAYS IN COVID‐19

The chronic inflammatory milieu in cardio‐metabolic disorders can induce the development of dysfunctional HDL, which in turn induces the release of pro‐inflammatory cytokines through activation macrophages.^70^, ^71^ High circulating dysfunctional HDL activates macrophage CD36 and suppresses expression of SR‐BI which reduces cholesterol efflux capacity and increases cholesterol deposition within the macrophages through a CD36‐dependent mechanism.^70^ Suppression and induction of SR‐BI and CD36 respectively by dysfunctional HDL are mediated by activation of MAPK and PPAR‐γ.^70^ In COVID‐19, high pro‐inflammatory cytokines may inhibit the expression of SR‐BI directly or through induction development of dysfunctional HDL which acts mainly on CD36 rather than SR‐BI. Cao et al. found that SARS‐CoV‐2 through direct activation of macrophages triggers upregulation of PPAR‐γ and MAPK^72^ which may affect the functional capacity of anti‐inflammatory SR‐BI in the lung. Though, PPAR‐γ modulates hepatic SR‐BI and thereby could be a protective mechanism against the progression of atherosclerosis in diabetic patients.^73^ MAPK affects the functional capacity of SR‐BI.^74^ Therefore, high MAPK and dysregulated PPAR‐γ could impair the function and expression of SR‐BI causing dyslipidemia and abnormal immune response.

During the pathogenesis of SARS‐CoV‐2 infection, node‐like receptor family pyrin domain‐containing protein 3 (NLRP3) inflammasome is activated with the release of inflammatory molecules and pro‐inflammatory cytokines.^75^ Mounting of innate immune response by activated NLRP3 inflammasome is associated with COVID‐19 severity by exaggeration of the immune response toward SARS‐CoV‐2 infection.^75^ It has been reported that activated NLRP3 inflammasome can inhibit the expression of SR‐BI, so inhibition of NLRP3 inflammasome improves uptake of cholesterol via upregulation of SR‐BI.^76^ Thus, NLRP3 inflammasome could be a potential link between the downregulation of SR‐BI and COVID‐19 severity.

Furthermore, high mobility group box 1(HMGB1) is regarded as a DAMP signal for TLR4 to trigger the release of pro‐inflammatory cytokines mainly IL‐6 and tumor necrosis factor (TNF‐α).^77^ HMGB1 is activated during SARS‐CoV‐2 infection leading to hyperinflammation and the development of cytokine storm.^78^ A retrospective study comprised 121 COVID‐19 patients, of whom 40 were severe compared to 81 mild ones, showed that HMGB1 serum level was higher in patients with severe COVID‐19.^78^ Extracellular HMGB1 promotes the translocation of LDL by enhancing the expression of endothelial SR‐BI causing endothelial dysfunction and the development of atherosclerosis.^79^ These findings proposed that HMGB1 overexpression in COVID‐19 might be a potential cause of endothelial injury through the LDL‐SR‐BI‐dependent pathway.

Of interest, the expression of NF‐κB during the inflammatory process inhibits the expression of SR‐BI.^80^ NF‐κB inhibitors like aspirin to improve the expression of SR‐BI and abrogate macrophage activation.^80^ In COVID‐19, NF‐κB is directly stimulated by SARS‐CoV‐2 leading to an exaggeration of the inflammatory process with the development of various complications.^81^ Thus, aspirin may be effective in COVID‐19 by reducing coagulopathy and inhibiting activated NF‐κB.^82^ These observations illustrated that exaggerated NF‐κB activity in SARS‐CoV‐2 infection could be a possible cause of the downregulation of SR‐BI in COVID‐19.

In the bargain, CD147 is regarded as a potential for binding and entry of SARS‐CoV‐2, and inhibition of CD147 by azithromycin may reduce the pathogenesis of SARS‐CoV‐2 infection.^83^ It has been reported that oxLDL stimulates the expression of CD147, while HDL inhibits its expression through the activation of SR‐BI.^84^ Interestingly, inhibition expression of CD147 through HDL‐SR‐BI may reduce CD147‐dependent SARS‐CoV‐2 entry and infection. Moreover, SR‐BI mediates the action of the protective effects of sphingosine‐1 phosphate (S1P) against endothelial dysfunction and dyslipidemia.^85^ Reduction of S1P serum level is linked with COVID‐19 severity.^86^ Therefore, the reduction of S1P in COVID‐19 could be due to the inhibition of SR‐BI by a high level of pro‐inflammatory cytokines.

**Figure 5:**
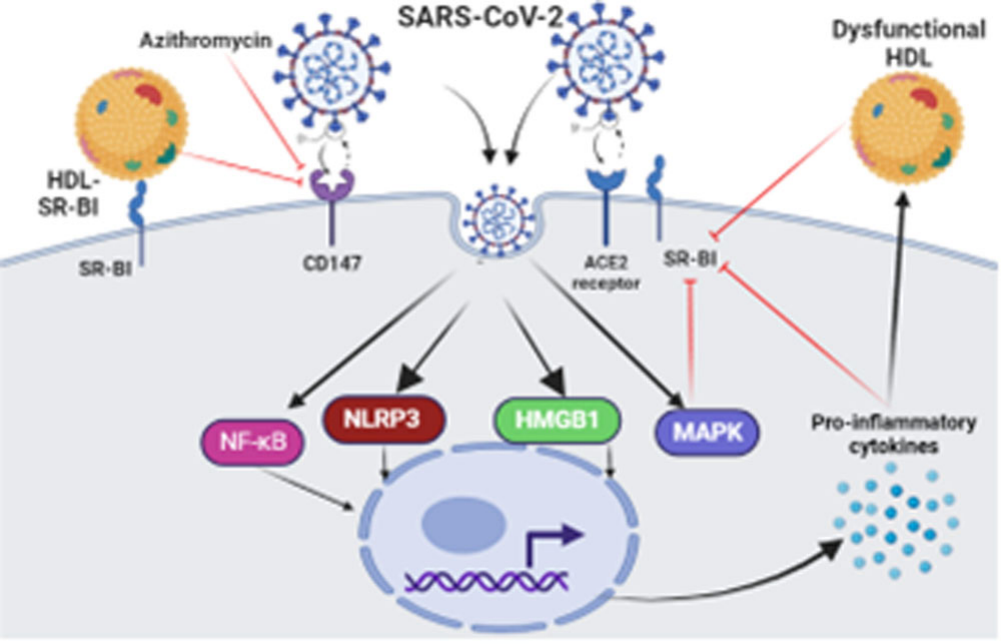
Inhibition of scavenger receptor type B I (SR‐BI) in COVID‐19. Severe acute respiratory syndrome coronavirus type 2 (SARS‐CoV‐2) through activation of node‐like receptor family pyrin domain‐containing protein 3 (NLRP3) inflammasome, nuclear factor kappa B (NF‐κB), high mobility group box 1(HMGB1) and mitogen‐activated protein kinase (MAPK) induces the release of pro‐inflammatory cytokines which inhibit SR‐BI. As well, pro‐inflammatory cytokines together with activation of MAPK and NLRP3 inflammasome induce the formation of dysfunctional HDL, which also inhibits the expression of SR‐BI.

These observations revealed that activated inflammatory signaling pathways during SARS‐CoV‐2 infection might be the underlying cause of dysfunctional SR‐BI in COVID‐19 (Figure 5). Further studies are requiring verifying the effects of these inflammatory signaling pathways on the expression and function of SR‐BI.

## 7 SR‐BI AND COAGULOPATHY IN COVID‐19

Severe SARS‐CoV‐2 infection is associated with profound coagulopathy including massive intravascular thrombosis and disseminated intravascular coagulation (DIC).^87^ COVID‐19‐induced coagulopathy is reflected by high d‐dimer which mirrors underlying fibrinogen/fibrin degradation products. The underlying mechanism of COVID‐19‐induced coagulopathy is not fully understood however, immune dysregulation, hypoxia, platelet hyperactivity, endothelial dysfunction, and pro‐inflammatory cytokines might be the proposed mechanisms.^87^ SR‐BI has a critical role in the regulation of coagulation homeostasis since deficiency of SR‐BI increases the risk of pulmonary embolism and deep vein thrombosis.^88^ SR‐BI improves endothelial NOS and release of NO which has anti‐atherogenic and anti‐thrombotic effects.^88^ As well, HDL‐SR‐BI controls endothelial function through the enhancement release of antithrombotic S1P and prostaglandin I2.^89^

SR‐BI is highly expressed on the platelets and plays an important role in the inhibition of platelet hyperreactivity through the reduction of cholesterol membrane content of platelets.^90^ Ma et al.^91^ observed that deficiency of platelet SR‐BI in mice increases platelet cholesterol content and hyper‐activity causing thrombosis. These findings revealed that SR‐BI may protect against thrombosis and cardiovascular complications in patients with dyslipidemia. In this sense, dyslipidemia has been shown to be correlated with COVID‐19 severity.^92^ Thus, abnormal function of SR‐BI due to abnormal inflammatory disorders in COVID‐19 might be a potential cause of dyslipidemia in COVID‐19.

Moreover, platelet‐mediated thrombotic events have been observed to induce immunomodulatory effects during influenza virus infection.^93^ Therefore, expression of SR‐BI could mitigate platelet‐induced abnormal immune response in COVID‐19. Furthermore, HDL through activation of SR‐BI induces fibrinolysis in patients with diabetes mellitus.^94^ Wright et al illustrated that the fibrinolytic pathway is highly inhibited in COVID‐19 and increases the risk of thromboembolic disorders.^95^

**Figure 6:**
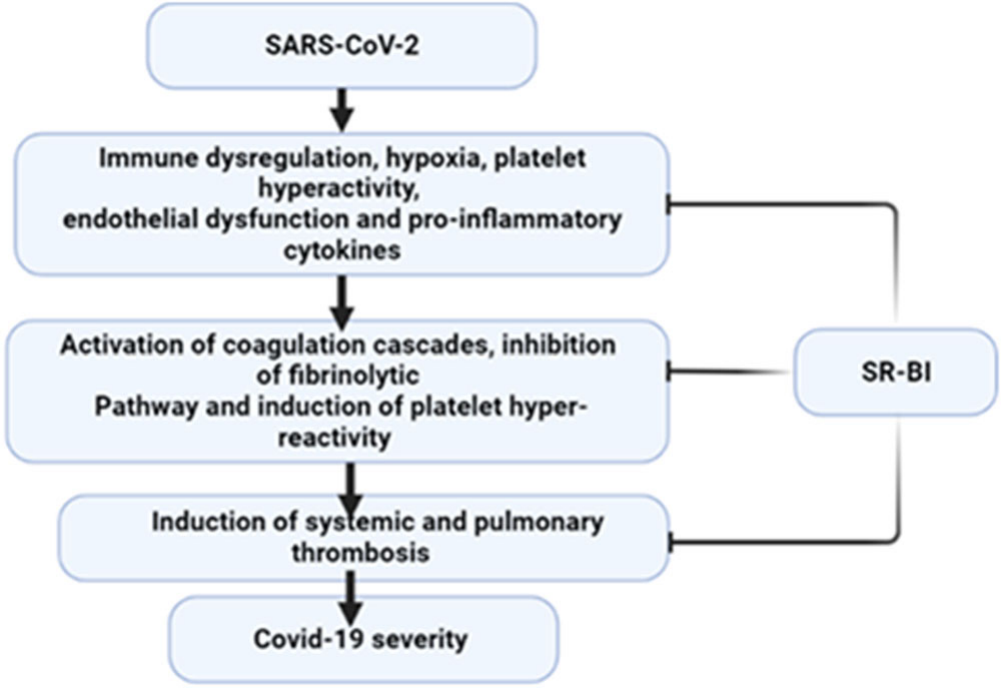
Role of scavenger receptor type B I (SR‐BI) and coagulopathy in COVID‐19.

Taken together, the expression of SR‐BI prevents thromboembolic disorders and other coagulopathy events in COVID‐19 through modulation of platelet function, coagulation, and fibrinolytic pathways (Figure 6).

## 8 SR‐BI AND ANGIOTENSIN II IN COVID‐19

AngII is upregulated in COVID‐19 due to the downregulation of ACE2 by SARS‐CoV‐2. High circulating AngII induces the release of pro‐inflammatory cytokines and the development of ALI/ARDS.^14^, ^15^ In addition, AngII in COVID‐19 can trigger endothelial dysfunction and vasoconstriction via inhibition of eNOS.^15^ Bian et al. found that AngII induces a pro‐atherogenic effect via provoking LDL transcytosis via endothelial barriers with induction formation of endothelial reactive oxygen species (ROS).^96^

It has been shown that AngII inhibits the expression of endothelial SR‐BI, and Ang II type 1 receptor (AT1R) blocker olmesartan can attenuate AngII‐induced downregulation of SR‐BI.^97^ The inhibitory effect of AngII on the expression of SR‐BI is mediated through the activation of phosphoinositide 3‐kinase (PI3K), protein kinase B (PKB) also known as Akt, and Forkhead Box O1 (FOXO1) pathways.^97^ Of note, AngII‐induced ALI/ARDS could be due to the downregulation expression of protective lung alveolar SR‐BI.^97^ It has been shown that Akt, PKB, and FOXO1 pathways are upregulated in COVID‐19 leading to endothelial dysfunction and metabolic disturbances.^98^ Normally, HDL inhibits the expression of AngII and AT1R, so attenuates AngII‐induced vascular inflammation.^45^, ^99^ In SARS‐CoV‐2 infection, HDL is reduced and correlated with COVID‐19 severity.^100^ Low HDL in this condition may facilitate the elevation of AngII level and the inhibitory effects on SR‐BI.

Of interest, AngII provokes the expression of vascular endothelial growth factor (VEGF) through the generation of ROS.^101^, ^102^ Dysregulated VEGF is highly activated in COVID‐19 and linked with neuroinflammation.^103^, ^104^ It has been disclosed that under normal conditions VEGF can regulate the localization of SR‐BI and trans‐endothelial transport of HDL.^68^ As well, VEGF improves the expression of SR‐BI, binding, and uptake of HDL in the endothelial cells.^68^, ^105^ Deregulated VEGF in COVID‐19 reduces the expression of SR‐BI and contributes to the development of dyslipidemia and the progression of endothelial dysfunction.^68^, ^106^

**Figure 7:**
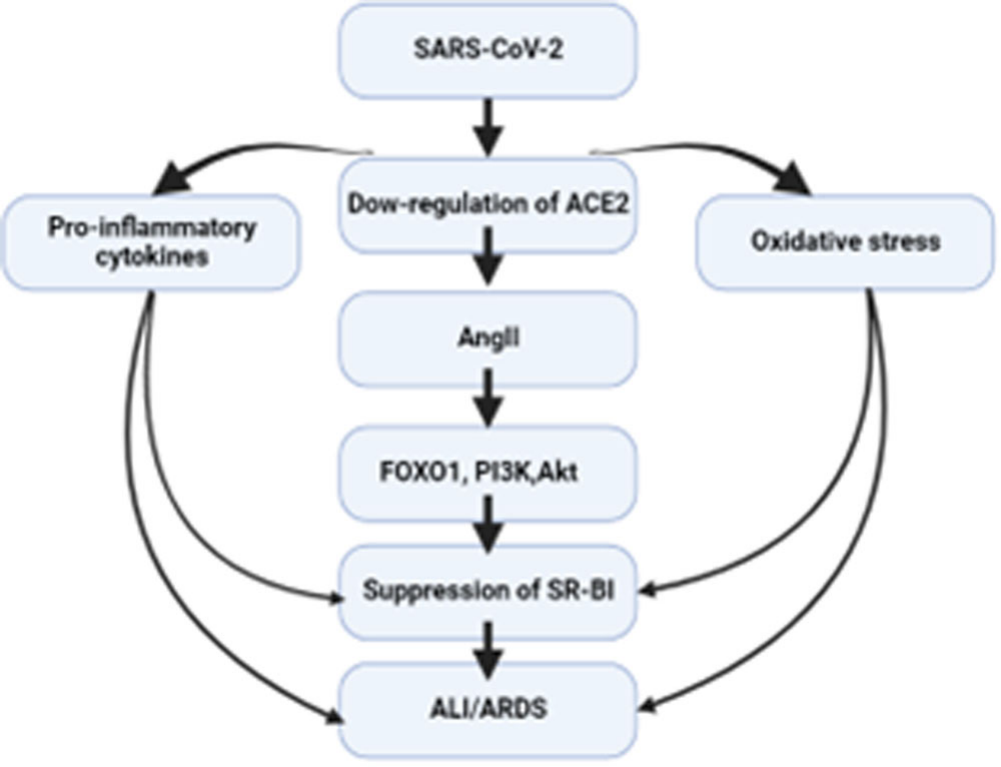
Interaction between scavenger receptor type B I (SR‐BI) and AngII in COVID‐19. Downregulation of ACE2 increases AngII which induces oxidative stress, the release of pro‐inflammatory cytokines, and activation of phosphoinositide 3‐kinase (PI3K), protein kinase B (PKB) also known Akt, and Forkhead Box O1 (FOXO1) pathways. These changes lead to the induction of acute lung injury (ALI) and acute respiratory distress syndrome (ARDS) with increasing COVID‐19 severity.

These verdicts indicate that high circulating AngII in COVID‐19 could be a possible cause of abnormal expression and activity of SR‐BI as well as dyslipidemia (Figure 7).

Taken together, SR‐BI is inhibited during COVID‐19 due to binding and consumption by SARS‐CoV‐2 infection. In addition, COVID‐19‐associated inflammatory changes and high AngII might be possible causes of repression of SR‐BI in SARS‐CoV‐2 infection.

## 9 CONCLUSIONS

SR‐BI is colocalized with ACE2 and controls immunoinflammatory disturbances during different viral infections. SR‐BI regulates innate immune response through the regulation of macrophage activity and proliferation and function of B and T lymphocytes. Inhibition of SR‐BI triggers the development of abnormal immune response and autoimmunity. SR‐BI is implicated as a receptor for entry of SARS‐CoV‐2 and in the pathogenesis of COVID‐19. Downregulation of SR‐BI in COVID‐19 could be due to direct invasion by SARS‐CoV‐2 or through upregulation of pro‐inflammatory cytokines, inflammatory signaling pathways, and high circulating AngII. Reduction of SR‐BI in COVID‐19 like ACE2 may provoke COVID‐19 severity through exaggeration of the immune response. Further studies are invoked to clarify the potential role of SR‐BI in the pathogenesis of COVID‐19 that could be protective rather than detrimental.

## AUTHOR CONTRIBUTIONS

**Luay Alkazmi**: Writing—review and editing. **Hayder M. Al‐kuraishy**: Conceptualization; methodology. **Ali I. Al‐Gareeb**: Data curation; resources; writing—original draft. **Athanasios Alexiou**: Supervision; validation; writing—review and editing. **Marios Papadakis**: Supervision; visualization; writing—review and editing. **Hebatallah M. Saad**: Data curation; resources; writing—original draft. **Gaber El‐Saber Batiha**: Supervision; visualization; writing—review and editing.

## CONFLICT OF INTEREST STATEMENT

The authors declare no conflict of interest.
